# SUCCINCT: An Open-label, Single-arm, Non-randomised, Phase 2 Trial of Gemcitabine and Cisplatin Chemotherapy in Combination with Sunitinib as First-line Treatment for Patients with Advanced Urothelial Carcinoma

**DOI:** 10.1016/j.eururo.2014.11.003

**Published:** 2015-04

**Authors:** Thomas Geldart, John Chester, Angela Casbard, Simon Crabb, Tony Elliott, Andrew Protheroe, Robert A. Huddart, Graham Mead, Jim Barber, Robert J. Jones, Joanna Smith, Robert Cowles, Jessica Evans, Gareth Griffiths

**Affiliations:** a Royal Bournemouth Hospital, Bournemouth, UK; b Institute of Cancer and Genetics, School of Medicine, Cardiff University, Cardiff, UK; c St James’ University Hospital, Leeds, UK; d Wales Cancer Trials Unit, School of Medicine, Cardiff University, Cardiff, UK; e University of Southampton Faculty of Medicine, Southampton General Hospital, Southampton, UK; f Christie Hospital, Manchester, UK; g Churchill Hospital, Oxford, UK; h Institute of Cancer Research and Royal Marsden Hospital, Surrey, UK; i Velindre Cancer Centre, Velindre Hospital, Cardiff, UK; j Institute of Cancer Sciences, University of Glasgow, Beatson West of Scotland Cancer Centre, Glasgow, UK

**Keywords:** Advanced urothelial tract transitional cell carcinoma, Phase 2, Clinical trial, First-line treatment, Sunitinib

## Abstract

Gemcitabine and cisplatin chemotherapy (GC regimen) represents a standard treatment for advanced urothelial carcinoma. We performed an open-label, single-arm, non-randomised, phase 2 trial evaluating the addition of sunitinib to standard GC chemotherapy (SGC regimen). Overall, 63 treatment-naïve participants were recruited and received up to six 21-d cycles of cisplatin 70 mg/m^2^ (intravenously [IV], day 1) and gemcitabine 1000 mg/m^2^ (IV, days 1 and 8) combined with sunitinib 37.5 mg (orally, days 2–15). Following review of toxicity after the first six patients, the sunitinib dose was reduced to 25 mg for all patients. Overall response rate was 64%, with response noted in 37 of 58 patients. At 6 mo, 30 of 58 assessable patients (52%; 90% confidence interval [CI], 40–63%) were progression free. Median overall survival was 12 mo (95% CI, 9–15) and was heavily influenced by Bajorin prognostic group. Grade 3–4 toxicities were predominantly haematologic and limited the deliverability of the triple SGC regimen. The trial did not meet its prespecified primary end point of >60% patients progression free at 6 mo. Cumulative myelosuppression led to treatment delays of gemcitabine and cisplatin and dose reduction and/or withdrawal of sunitinib in the majority of cases. The triple-drug combination was not well tolerated. Phase 3 evaluation of the triple SGC regimen in advanced transitional cell carcinoma is not recommended.

**Patient summary:**

The addition of sunitinib to standard cisplatin and gemcitabine chemotherapy was poorly tolerated and did not improve outcomes in advanced urothelial carcinoma. Treatment delivery was limited by myelotoxicity.

The prognosis for patients with advanced urothelial carcinoma is poor, and in the United Kingdom, approximately 5000 patients die each year from this disease [1]. Combination gemcitabine and cisplatin chemotherapy (GC regimen) represents a current standard of care in this disease setting, with randomised controlled trial evidence demonstrating progression-free survival (PFS) of 7 mo and overall survival (OS) of 14 mo in the first-line setting [2].

Novel targeted agents have led to significant improvements in outcome for patients with a wide variety of malignancies, but there have been few studies in advanced urothelial cancer. Sunitinib, an oral multitargeted-receptor tyrosine kinase inhibitor, has potent antiangiogenic and antitumour activity. Microvessel density (a measure of tumour angiogenesis) and high serum vascular endothelial growth factor (VEGF) levels appear to be associated with a poorer outcome in urothelial carcinoma and, in particular, may be associated with higher disease stage, higher grade, vascular invasion, and poorer disease-free survival [3], [4]. Preclinical and early phase clinical studies confirmed activity of sunitinib in urothelial cancer and showed that it could be combined with GC cytotoxic chemotherapy [5], [6], [7].

In this open-label, single-arm, non-randomised, phase 2 trial, we evaluated the addition of sunitinib to standard GC chemotherapy (SGC regimen; detailed inclusion criteria, efficacy assessments, and statistical considerations are shown in the supplementary data). Eligibility criteria included patients with World Health Organisation performance status of 0–2 and advanced, histologically confirmed urothelial (transitional cell) carcinoma who were fit enough to receive cisplatin-containing chemotherapy. All patients received up to six 21-d cycles of GC chemotherapy (cisplatin 70 mg/m^2^ intravenously [IV] on day 1, gemcitabine 1000 mg/m^2^ IV on days 1 and 8) in combination with sunitinib 37.5 orally each day on days 2–15. Following review of haematologic toxicity after enrolment of the first six patients, sunitinib dose was reduced to 25 mg orally each day on days 2–15 for all patients.

The primary end point of the study was PFS at 6 mo. The sample size of 63 was based on Fleming's one-stage design using a significance level (one-sided) of 10% and 90% power. The expected PFS at 6 mo following treatment with standard GC chemotherapy was approximately 65% [2]. PFS at 6 mo of <60% was deemed to be insufficiently large enough to warrant further investigation. Secondary end points included time-to-event analysis of PFS and OS, safety, tolerability, and objective overall response rate (ORR).

Between 31 July 2009 and 1 February 2013, 63 patients were recruited from 11 institutions in the United Kingdom (patient characteristics and CONSORT diagram are shown in Fig. 1; supplementary data). Overall, 58 patients were included in the analysis of PFS and ORR. All 63 patients were included in the secondary analyses.

Treatment-related outcomes are summarised in Table 1 and Figure 1. Patients received a median of six cycles of treatment (interquartile range: 3–6). Moreover, 21% (12 of 58 patients) achieved complete radiologic response, 43% (25 of 58) achieved partial response, and 14% (8 of 58) achieved stable disease, for an ORR of 64% and a disease control rate of 78%.

At 6 mo, 52% (30 of 58 patients) remained progression free (90% confidence interval [CI], 40–63%). For the time-to event-analysis, the median PFS for all patients was 8 mo (95% CI, 6–11). A total of 39 patients (62%) died of progressive disease. Median OS was 12 mo (95% CI, 9–15).

Table 2 summarises all reported, treatment-related, Common Terminology Criteria for Adverse Events grade 3–4 toxicities occurring in >5% of patients during treatment. Reported toxicities were predominantly haematologic. Prolonged myelosuppression was common. Despite a reduction in the starting dose of sunitinib from 37.5 mg to 25 mg, the majority of patients required further sunitinib dose reduction or withdrawal for a variety of reasons including intolerance of treatment (*n* = 18), clinician choice (*n* = 11), disease progression (*n* = 5), patient choice (*n* = 2), poor performance status (*n* = 1), and bowel obstruction (*n* = 1). Nonhaematologic toxicities were infrequently reported, with grade 3–4 fatigue occurring in five patients (8%) and gastrointestinal toxicity (nausea, vomiting, and diarrhoea, combined) in seven patients (11%). By cycle 6, only 33% of patients remained on full dose sunitinib; cisplatin and gemcitabine doses were well preserved, but dose delay was common. Relative dose intensity fell with successive cycles of treatment (Figure 2 and Table 2, supplementary data).

There was no evidence that treatment outcomes were improved following the addition of sunitinib. The triple SGC regimen was associated with high levels of haematologic toxicity and dose delay. Response rate was in keeping with that expected for GC alone, and no improvement was found in PFS or OS following the addition of sunitinib. OS was heavily influenced by Bajorin risk group [8]. No evidence showed that sunitinib improved outcome in any subgroup, although the number of patients with poor-prognosis disease was small (Table 1, Fig. 1C).

The combination of sunitinib with standard cytotoxic chemotherapy appears to prolong the duration of myelosuppression seen with standard cytotoxic chemotherapy. Although myelotoxicity is seen with single-agent sunitinib, this is rarely dose limiting, with grade 3–4 toxicity occurring in <10% of patients. The toxicities and outcomes seen in our study are in keeping with updated results from the original phase 1 SGC study in lung cancer and two smaller contemporaneous studies that sought to combine standard cytotoxic chemotherapy with sunitinib in patients with urothelial carcinoma [9], [10]. The synergistic myelosuppressive effects of sunitinib may relate to inhibition of receptor tyrosine kinases other than VEGF, and it may be that these “off-target” effects of sunitinib are important for bone marrow recovery following standard cytotoxic chemotherapy. Given the potentially important role of angiogenesis in the development and progression of advanced urothelial cancer, alternative strategies for targeting the VEGF pathway may prove more fruitful. A large phase 3 trial is currently under way to evaluate standard GC chemotherapy with or without bevacizumab in the treatment of advanced transitional cell carcinoma (ClinicalTrials.gov identifier NCT00942331).

In conclusion, the addition of sunitinib to standard-dose GC chemotherapy was not well tolerated, and no evidence showed improved outcomes for patients with advanced urothelial carcinoma. Treatment was limited by cumulative myelotoxicity. These results are in keeping with clinical trials using sunitinib and cytotoxic chemotherapy combinations in other solid tumours. The triple SGC combination is not recommended for further phase 3 evaluation in patients with advanced urothelial carcinoma.

  ***Author contributions:*** Angela Casbard had full access to all the data in the study and takes responsibility for the integrity of the data and the accuracy of the data analysis.  

*Study concept and design:* Geldart, Chester, Casbard, Mead, Griffiths.

*Acquisition of data:* Jones, Crabb, Elliott, Protheroe, Huddart, Mead, Chester, Barber, Geldart.

*Analysis and interpretation of data:* Casbard, Evans, Geldart, Griffiths.

*Drafting of the manuscript:* Geldart, Casbard, Griffiths.

*Critical revision of the manuscript for important intellectual content:* None.

*Statistical analysis:* Casbard, Evans.

*Obtaining funding:* Geldart, Chester, Casbard, Mead, Griffiths.

*Administrative, technical, or material support:* Cowles, Smith.

*Supervision:* None.

*Other* (specify): None.  

***Financial disclosures:*** Angela Casbard certifies that all conflicts of interest, including specific financial interests and relationships and affiliations relevant to the subject matter or materials discussed in the manuscript (eg, employment/affiliation, grants or funding, consultancies, honoraria, stock ownership or options, expert testimony, royalties, or patents filed, received, or pending), are the following: Thomas Geldart has received a Pfizer travel grant and speaker fee and a Boehringer Ingelheim/Novartis travel grant. Gareth Griffiths has previously acted as an independent statistical consultant on trial designs to Pfizer and as a director of an academic clinical trials unit in the United Kingdom, and he has received numerous academic investigator-initiated research funding awards (educational grants) and free drugs from Pfizer for numerous cancer areas under the banner of the UK National Cancer Research Institute.  

***Funding/Support and role of the sponsor:*** The trial was funded by Cancer Research UK (CRUK; Clinical Trials Awards and Advisory Committee grant A9325/C9347) and CRUK core funding at the Wales Cancer Trials Unit. These sponsors were involved in the design and conduct of the study; the collection, management, analysis, and interpretation of the data; and the preparation, review, and approval of the manuscript. Pfizer provided free sunitinib, labelling, and distribution for the study and a limited research grant but had no influence on how we designed, ran, and reported the trial and had no access to any data.  

***Acknowledgment statement:*** The SUCCINCT trial was developed on behalf of the UK National Cancer Research Institute Bladder Cancer Clinical Studies Group and sponsored by Cardiff University. We thank all the participants, the doctors, UK National Institute for Health Research Clinical Research Network Cancer research nurses, and other members of the multidisciplinary teams and research teams who supported this trial at participating centres. We thank members of the independent data monitoring committee (Jeff Evans, Emma Hall, and Ruth Plummer) and the independent trial steering committee (Barry Hancock, John Wagstaff, and Stephen Shepherd) for their oversight of the trial. We also thank Sally Munden and Alison Hogan (trial pharmacists), Philip Bell and Colin Thompson (patient representatives), Lynette Lane (nursing advisor), and Margaret Knowles for their input into the trial management group.

## Figures and Tables

**Fig. 1:**
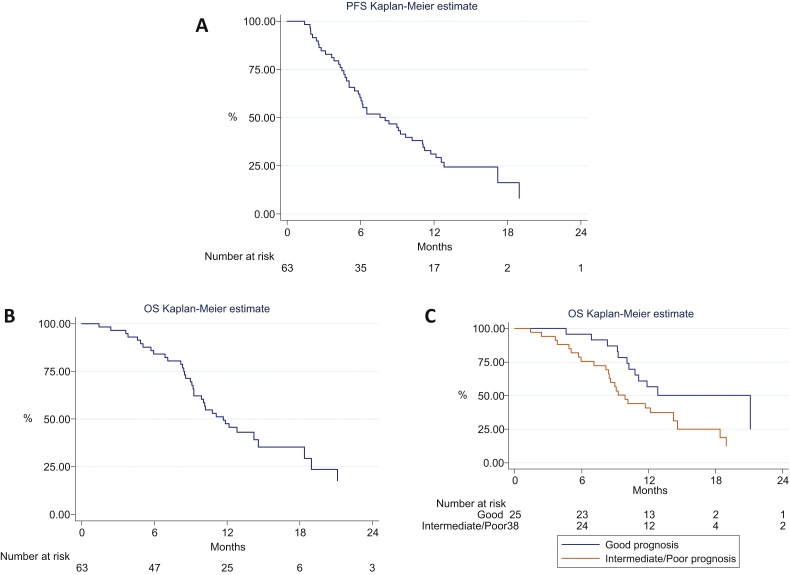
(A) Progression-free survival, (B) overall survival (OS), and (C) OS by Bajorin prognostic group.

**Fig. 2:**
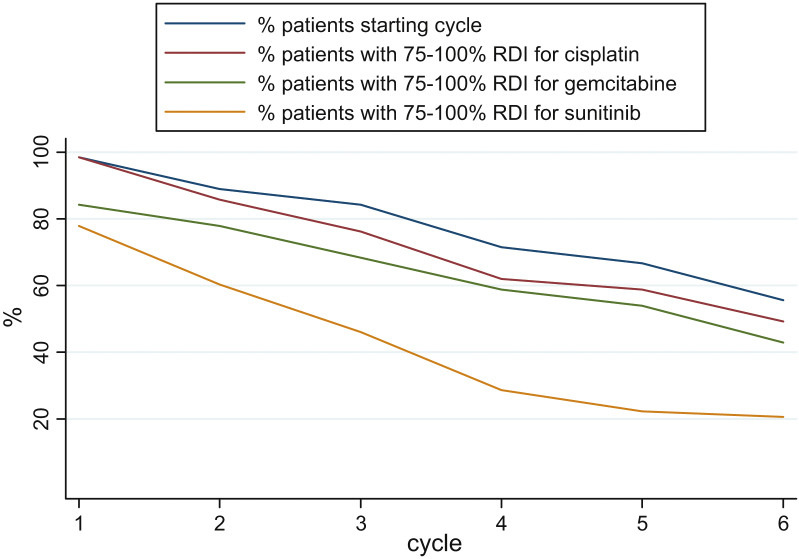
Relative dose intensity (actual dose intensity divided by expected dose intensity) by cycle and treatment. RDI = relative dose intensity.

**Table 1:** Overall outcome

Outcome	Results
**Primary end point**	
6-mo PFS (*n* = 58*), % (90% CI)	52 (40–63)
**Secondary end points**	
Overall response rate (*n* = 58), *n* (%)	37 (64)
CR	12 (21)
PR	25 (43)
SD	8 (14)
Disease control, CR + PR + SD	45 (78)
Time-to-event PFS (*n* = 63), mo, median (95% CI)	8 (6–11)
Time-to-event OS (*n* = 63), mo, median (95% CI)	12 (9–15)
Time-to-event OS by Bajorin prognostic group, mo, median (95% CI)	
Good prognosis (*n* = 25)	21 (10–NR)
Intermediate prognosis (*n* = 36)	10 (8–14)
Poor prognosis (*n* = 2)	4 (4–NR)

* Five of 63 patients withdrew prior to response assessment. CI = confidence interval; CR = complete response; NR = not reached; OS = overall survival; PFS = progression-free survival; PR = partial response; SD = stable disease.

**Table 2:** Treatment-related toxicity (grade ≥3) occurring in ≥5% of patients in one cycle or more of treatment

Toxicity	Worst grade reported, *n* (%)		
	3	4	3 or 4
Overall worst grade per patient (any toxicity)	18 (28.6)	36 (57.1)	54 (85.7)
Anaemia	15 (23.8)	1 (1.6)	16 (25.4)
Leukopenia	32 (50.8)	8 (12.7)	40 (63.5)
Neutropenia	16 (25.4)	31 (49.2)	47 (74.6)
Thrombocytopenia	21 (33.3)	12 (19.0)	33 (52.3)
Neutropenic fever or sepsis	4 (6.3)	3 (4.8)	7 (11.1)
Combined GI toxicity (nausea, vomiting, or diarrhoea)	7 (11.1)	0 (0.0)	7 (11.1)
Fatigue	5 (7.9)	0 (0.0)	5 (7.9)

GI = gastrointestinal.

## Appendix A

The following institutions and clinicians participated in the trial: Addenbrooke's Hospital (Danesh Mazhar), Beatson West of Scotland Cancer Centre (Robert J. Jones), Christie Hospital (Tony Elliott), Churchill Hospital (Andrew Protheroe), Royal Bournemouth Hospital (Thomas Geldart), Royal Marsden (Robert A. Huddart), Royal Shrewsbury Hospital (Narayanan Srihari), Southampton General Hospital (Graham Mead, Simon Crabb), St Barts Hospital (Tom Powles), St James's University Hospital (John Chester), Velindre Hospital (Jim Barber).
