# Supplementary material for: SUCCINCT: An Open-label, Single-arm, Non-randomised, Phase 2 Trial of Gemcitabine and Cisplatin Chemotherapy in Combination with Sunitinib as First-line Treatment for Patients with Advanced Urothelial Carcinoma
Source: Eur Urol. 2015 Apr;67(4):599–602. doi: 10.1016/j.eururo.2014.11.003 (PMC4410296; doi:10.1016/j.eururo.2014.11.003)
Supplement: Supplementary file 1 [file mmc1.doc]

**Supplementary data – Patients and methods**

The SUCCINCT trial (Sunitinib in advanced Urothelial CanCer IN Combination with standard cisplatin/gemcitabine chemotherapy Treatment; EudraCT no. 2007-007591-42; ISRCTN 54607216) was approved by the UK Medicines and Healthcare Products Regulatory Agency and a multicentre research ethics committee. All patients provided written informed consent at enrolment.

***Eligibility***

Main inclusion criteria included (1) histologically confirmed urothelial (upper or lower urinary tract) transitional cell carcinoma (pure or mixed histology); (2) radiologically measurable, locally advanced, and/or metastatic disease not amenable to curative treatment with surgery or radiotherapy (ie, T4b [bladder] or T4 [renal pelvis or ureter], N_any_, M_any_; T_any_N2–3M_any_; or T_any_N_any_M1); (3) estimated life expectancy ≥3 mo; (4) age ≥16 yr; (5) World Health Organisation performance status 0–2; (6) fit to receive cisplatin-containing combination chemotherapy; (7) no prior systemic therapy for locally advanced or metastatic disease, with prior neoadjuvant or adjuvant chemotherapy for urothelial cancer (up to four cycles) completed at least 6 mo prior to first documented disease progression permissible; (8) no prior radiotherapy within 1 mo of registration or involving >30% of total bone marrow volume; (9) adequate renal function (glomerular filtration rate >60 ml/min), bone marrow function (absolute neutrophil count ≥1.5 × 10^9^ per litre; platelets ≥100 × 10^9^ per litre), and liver function (bilirubin ≤1.5 times the upper limit of normal [ULN]; alanine aminotransferase and alkaline phosphatase ≤2.5 times ULN).

***Treatment***

Patients started treatment within 2 wk of the date of enrolment. All patients received up to six 21-d cycles of gemcitabine and cisplatin (GC regimen) chemotherapy in combination with sunitinib (SGC regimen). Treatment comprised cisplatin (70 mg/m^2^ intravenously [IV]) on day 1, gemcitabine (1000 mg/m^2^ IV) on days 1 and 8, and sunitinib 37.5 mg orally each day on days 2–15. Following review of toxicity after enrolment of the first six patients, sunitinib dose was reduced to 25 mg orally each day on days 2–15 for the subsequent 57 patients. Full-dose cisplatin and gemcitabine were maintained throughout the trial.

***Assessment of efficacy and safety***

Restaging computed tomography scans were performed at weeks 9, 17, 26, and 52 to assess disease response to treatment and were reported according to Response Evaluation Criteria in Solid Tumours (RECIST) v1.0. Clinical evaluation, haematology, and biochemistry were performed prior to and during each treatment cycle. Nuclear medicine bone scans and cystoscopy were performed as clinically relevant. Toxicity data during and after treatment were categorised using US National Cancer Institute Common Terminology Criteria for Adverse Events (CTCAE) v3.0 with real-time reporting of serious adverse events.

***Statistical analysis***

The primary end point of the study was progression-free survival (PFS) at 6 mo. The sample size of 63 was based on Fleming’s one-stage design using a significance level (one-sided) of 10% and 90% power. On the basis of previously published results, the expected PFS at 6 mo in this patient group treated with GC chemotherapy was approximately 65% [1]. A PFS at 6 mo of <60% (p1) was deemed to be insufficiently large enough to warrant further investigation. A PFS of ≥75% (p2) was deemed sufficient to support consideration of further investigation in a phase 3 setting. The primary end point was selected as the proportion of patients alive and free from RECIST-defined disease progression at 6 mo, with the corresponding 90% confidence interval [CI]. Because Fleming’s design uses a binary end point at the time point of 6 mo, we allowed a window of 4 wk on either side of the 6-mo RECIST evaluation (ie, 22–30 wk), with all death or RECIST-confirmed disease progression before 6 mo considered as events at 6 mo. Secondary end points included time-to-event analysis of PFS and overall survival (OS), safety, and objective overall response rate. Survival estimates were summarised using medians and 95% CIs. Continuous variables were summarised using medians and interquartile ranges. The distribution of each categorical variable was summarised by its frequencies and percentages with 95% CIs. PFS was defined as the time from enrolment to evidence of RECIST disease progression or death from any cause. Patients alive and with no evidence of radiologic disease progression were censored at the time of last follow-up. OS was defined as the time from randomisation to death from any cause. Patients still alive were censored at the time of last follow-up. Kaplan-Meier curves were used to estimate unadjusted PFS and OS time distributions. Overall response rate was defined as the percentage of patients achieving a complete or partial response by RECIST criteria and is presented at a percentage with 95% CI. A subgroup analysis based on Bajorin prognostic categories was undertaken [2]. Data were analysed with the Stata11 statistical package.

***Patient characteristics***

Between 31 July 2009 and 1 February 2013, 63 patients were recruited from 11 institutions in the United Kingdom (CONSORT diagram; Supplementary Fig. 1). After recruitment, six participants totally withdrew consent for future data collection. One participant who withdrew was included in the primary analysis because the patient progressed before the time of withdrawal. Hence, a total of 58 patients were included in the analysis of the primary end point and overall response rate. All 63 patients were included in the secondary analyses. Patient characteristics are summarised in Supplementary Table 1.

***Supplementary discussion***

Patient outcomes following systemic treatment have not improved meaningfully since the initial publications evaluating (MVAC regimen) chemotherapy in the early 1990s and subsequent development of the GC regimen [3–9]. The addition of other active cytotoxic agents such as paclitaxel to standard GC chemotherapy or intensification of MVAC with the addition of growth factors has not resulted in statistically significant improvements in survival, and a plateau of effectiveness for standard cytotoxic chemotherapy seems to have been reached [10].

At the time this trial was conceived (in 2007), phase 1 data presented in abstract form suggested that GC chemotherapy administered to patients with lung cancer on the same schedule (but at doses in excess of those standardly used for the treatment of advanced urothelial cancer: cisplatin 80 mg/m^2^ and gemcitabine 1250 mg/m^2^) could be safely combined with sunitinib 37.5mg daily on a schedule of 2 wk on, 1 wk off in patients with advanced lung cancer [11].

We initially set out to evaluate the 3-wk combination of cisplatin given at a standard dose of 70 mg/m^2^ on day 1, gemcitabine given at 1000 mg/m^2^ IV on days 1 and 8, and sunitinib given at 37.5 mg orally each day on days 2–15.

A significant finding of this study is that the SGC drug triplet was not well tolerated even after sunitinib dose reduction. The predominant toxicity throughout the study was haematologic. For the majority of patients, it was not possible to maintain adequate sunitinib dose intensity, and most patients required dose reduction and/or discontinuation of this drug with increasing numbers of treatment cycles. Although cisplatin and gemcitabine doses were well preserved for patients remaining on treatment throughout their course of treatment, dose intensity was not, as the result of dose delays secondary to prolonged myelosuppression.

Cumulative myelosuppression in the first six patients in this study resulted in higher-than-expected dose delays and dose reductions, and updated data from the final publication of the phase 1 SGC triplet in lung cancer [12] demonstrated cumulative myelotoxicity (beyond cycle 1) to be greater than that seen in the original abstract. On those bases, we decided to reduce the dose of sunitinib from 37.5 mg to 25 mg orally on days 2–15, while maintaining full doses of cisplatin and gemcitabine, for the remainder of the trial.

Despite substantial dose reduction, the combination of sunitinib with standard-dose cytotoxic chemotherapy appears to unacceptably prolong the duration of myelosuppression experienced by patients. Updated information, published in 2010 in the final manuscript of the original phase 1 trial in lung cancer, suggests that this effect did not appear to be pharmacokinetic, as there was no significant change in C_max_ and AUC_0-24_ when the drugs were administered alone or in combination. The myelosuppressive effects of sunitinib may relate to inhibition of receptor tyrosine kinases other than VEGF, such as c-KIT and FLT3, and it may be that these off-target effects of sunitinib are important for bone marrow recovery following standard cytotoxic chemotherapy.

Potential limitations of our study include the single-arm design and the fact that only 58 of 63 patients were eligible for final analysis of the primary end point. Study outcomes may have been influenced by selection, although unacceptable toxicity limiting deliverability of the three-drug regimen would still have been a key finding had we performed a larger or two-arm randomised study.

Our results are in keeping with the toxicities and outcomes seen in other studies across a variety of tumour types that have sought to combine standard cytotoxic chemotherapy with sunitinib [13–15]. In urothelial cancer, two small studies that were performed contemporaneously in the United States evaluated the same three-drug SGC combination in patients with urothelial cancer, as neoadjuvant or palliative treatment. Patients were treated with a lower dose of standard GC chemotherapy, and neither trial completed accrual. A combined report of data from patients treated in the two trials revealed the emergence of similar dose-limiting myelosuppression with no improvement in outcome [16].

Given the potentially important role of angiogenesis in the development and progression of advanced transitional cell carcinoma (TCC), alternative strategies for targeting the VEGF pathway may prove more fruitful and easier to deliver than the combination of a tyrosine kinase inhibitor with cytotoxic chemotherapy. Phase 2 trials evaluating the addition of the nonmyelosuppressive anti-VEGF monoclonal bevacizumab to cisplatin- or carboplatin-based chemotherapy have produced encouraging results with acceptable toxicity [17–19]. A large phase 3 trial is currently under way to evaluate standard GC chemotherapy with or without bevacizumab in the treatment of advanced TCC (ClinicalTrials.gov identifier NCT00942331).

**References**

[1] von der Maase H, Hansen SW, Roberts JT, et al. Gemcitabine and cisplatin versus methotrexate, vinblastine, doxorubicin, and cisplatin in advanced or metastatic bladder cancer: results of a large, randomized, multinational, multicenter, phase III study. J Clin Oncol 2000;18:3068–77.

[2] Bajorin DF, Dodd PM, Mazumdar M, et al. Long-term survival in metastatic transitional-cell carcinoma and prognostic factors predicting outcome of therapy. J Clin Oncol 1999;17:3173–81.

[3] Sternberg CN, Yagoda A, Scher HI, et al. Preliminary results of M-VAC (methotrexate, vinblastine, doxorubicin and cisplatin) for transitional cell carcinoma of the urothelium. J Urol 1985;133:403–7.

[4] Harker WG, Meyers FJ, Freiha FS, et al. Cisplatin, methotrexate, and vinblastine (CMV): an effective chemotherapy regimen for metastatic transitional cell carcinoma of the urinary tract. A Northern California Oncology Group Study) . J Clin Oncol 1985;3:1463–70.

[5] Logothetis CJ, Dexeus FH, Finn L, et al. A prospective randomized trial comparing M-VAC and CISCA chemotherapy for patients with metastatic urothelial tumors. J Clin Oncol 1990;8:1050–55.

[6] Loehrer PJ Sr, Einhorn LH, Elson PJ, et al. A randomized comparison of cisplatin alone or in combination with methotrexate, vinblastine, and doxorubicin in patients with metastatic urothelial carcinoma: a cooperative group study) . J Clin Oncol 1992;10:1066–73.

[7] Sternberg CN, de Mulder PH, Schornagel JH, et al; European Organization for Research and Treatment of Cancer Genitourinary Tract Cancer Cooperative Group. Randomized phase III trial of high-dose-intensity methotrexate, vinblastine, doxorubicin, and cisplatin (MVAC) chemotherapy and recombinant human granulocyte colony-stimulating factor versus classic MVAC in advanced urothelial tract tumors: European Organization for Research and Treatment of Cancer Protocol no. 30924. J Clin Oncol 2001;19:2638–46.

[8] Moore MJ, Winquist EW, Murray N, et al. Gemcitabine plus cisplatin, an active regimen in advanced urothelial cancer: a phase II trial of the National Cancer Institute of Canada Clinical Trials Group. J Clin Oncol 1999;17:2876–81.

[9] Von der Maase H, Sengelov L, Roberts JT, et al. Long-term survival results of a randomized trial comparing gemcitabine plus cisplatin, with methotrexate, vinblastine, doxorubicin plus cisplatin in patients with bladder cancer. J Clin Oncol 2005;23:4602–8.

[10] Bellmunt J, von der Maase H, Mead GM, et al. Randomized phase III study comparing paclitaxel/cisplatin/gemcitabine and gemcitabine/cisplatin in patients with locally advanced or metastatic urothelial cancer without prior systemic therapy: EORTC Intergroup Study 30987. J Clin Oncol 2012;30:1107–13.

[11] Reck M, Frickhofen N, Gatzemeier U, et al. A phase I dose escalation study of sunitinib in combination with gemcitabine + cisplatin for advanced non-small cell lung cancer (NSCLC) [abstract]. J Clin Oncol 2007;25(Suppl):18057.

[12] Reck M, Frickhofen N, Cedres S, et al. Sunitinib in combination with gemcitabine plus cisplatin for advanced non-small cell lung cancer: a phase I dose-escalation study. Lung Cancer 2010;70:180–7.

[13] Wang Z, Wang M, Yang F, et al. Multitargeted antiangiogenic tyrosine kinase inhibitors combined to chemotherapy in metastatic breast cancer: a systematic review and meta-analysis. Eur J Clin Pharmacol 2014;70:531–8.

[14] Crown JP, Diéras V, Staroslawska E, et al. Phase III trial of sunitinib in combination with capecitabine versus capecitabine monotherapy for the treatment of patients with pretreated metastatic breast cancer. J Clin Oncol 2013;31:2870–8.

[15] Heist RS, Wang X, Hodgson L, et al. CALGB 30704 (Alliance): a randomized phase II study to assess the efficacy of pemetrexed or sunitinib or pemetrexed plus sunitinib in the second-line treatment of advanced non-small-cell lung cancer. J Thorac Oncol 2014;9:214–21.

[16] Galsky MD, Hahn NM, Powles T, et al. Gemcitabine, cisplatin, and sunitinib for metastatic urothelial carcinoma and as preoperative therapy for muscle-invasive bladder cancer. Clin Genitourin Cancer 2013;11:175–81.

[17] Hahn NM, Stadler WM, Zon RT, et al. Phase II trial of cisplatin, gemcitabine, and bevacizumab as first-line therapy for metastatic urothelial carcinoma: Hoosier Oncology Group GU 04-75. J Clin Oncol 2011;29:1525–30.

[18] Balar AV, Apolo AB, Ostrovnaya I, et al. Phase II study of gemcitabine, carboplatin, and bevacizumab in patients with advanced unresectable or metastatic urothelial cancer. J Clin Oncol 2013;31:724–30.

[19] Siefker-Radtke AO, Kamat AM, Corn PG, et al. Neoadjuvant chemotherapy with DD-MVAC and bevacizumab in high-risk urothelial cancer: results from a phase II trial at the University of Texas M. D. Anderson Cancer Center [abstract 4523]. J Clin Oncol 2012;30(Suppl):282s.
