# Supplementary material for: SUCCINCT: An Open-label, Single-arm, Non-randomised, Phase 2 Trial of Gemcitabine and Cisplatin Chemotherapy in Combination with Sunitinib as First-line Treatment for Patients with Advanced Urothelial Carcinoma
Source: Eur Urol. 2015 Apr;67(4):599–602. doi: 10.1016/j.eururo.2014.11.003 (PMC4410296; doi:10.1016/j.eururo.2014.11.003)
Supplement: Supplementary file 2 [file mmc2.doc]

**Supplementary Table 1 – Patient characteristics**

|  | ***n*** | **%** |
| --- | --- | --- |
| **Sex**  Male  Female | 56  7 | 89  11 |
| **Age**, yr, median (IQR) | 64 (56–68) | |
| **WHO PS**  0  1  2 | 42  19  2 | 67  30  3 |
| **Location of primary disease**  Bladder  Ureter  Renal pelvis | 52  6  5 | 82.5  9.5  8 |
| **Histology**  Pure TCC  Mixed | 55  8 | 87  13 |
| **Grade**  1  2  3  4 | 1  8  53  1 | 1.5  13  84  1.5 |
| **T stage**  TX  <T2  T2  T3  T4 | 7  13  21  11  11 | 11  21  33  17.5  17.5 |
| **N stage**  N0  N1  N2  N3 | 13  11  31  8 | 21  17  49  13 |
| **M stage**  M0  M1  Nonvisceral  Visceral only  Both | 11  52  14  16  22 | 17  83  22  25  35 |
| **Visceral sites of metastatic disease  (*n* = 38)**  Including lung  Including bone  Including liver  Including other sites | 21  9  7  11 | 55  24  18  29 |
| **Bajorin prognostic group**  Good  Intermediate  Poor | 25  36  2 | 39  57  3 |

IQR = interquartile range; TCC = transitional cell carcinoma; WHO PS = World Health Organisation performance status.

**Supplementary Table 2 – Treatment dose intensity and delay by cycle**

|  | Treatment | Cycle 1 | Cycle 2 | Cycle 3 | Cycle 4 | Cycle 5 | Cycle 6 |
| --- | --- | --- | --- | --- | --- | --- | --- |
| Full dose delivered, %, median (IQR) | Cisplatin | 100 (100–100) | 100 (100–100) | 100 (100–100) | 100 (100–100) | 100 (100–100) | 100 (100–100) |
|  | Gemcitabine | 100 (100–100) | 100 (100–100) | 100 (75–100) | 100 (75–100) | 84.9 (75–100) | 100 (75–100) |
|  | Sunitinib | 100 (92.9–100) | 100 (59.5–100) | 92.9 (42.9–100) | 50 (0–100) | 46.4 (0–100) | 33.3 (0–100) |
| Patients delayed/starting cycle, n (%) | SGC | 0/62 (0) | 39/56 (69.6) | 35/53 (66.0) | 26/45 (57.8) | 26/42 (61.9) | 19/35 (54.3) |
| Delay, d, median (IQR) | SGC | NA | 7 (6–9) | 8 (7–14) | 7 (5–14) | 7 (7–9) | 7 (7–14) |

IQR = interquartile range; NA = not available; SGC = sunitinib added to the gemcitabine and cisplatin regimen.
