# Supplementary material for: SUCCINCT: An Open-label, Single-arm, Non-randomised, Phase 2 Trial of Gemcitabine and Cisplatin Chemotherapy in Combination with Sunitinib as First-line Treatment for Patients with Advanced Urothelial Carcinoma
Source: Eur Urol. 2015 Apr;67(4):599–602. doi: 10.1016/j.eururo.2014.11.003 (PMC4410296; doi:10.1016/j.eururo.2014.11.003)
Supplement: Supplementary file 3 [file mmc3.doc]

**Supplementary Fig. 1 – CONSORT diagram.**

Excluded (*n* = 72)

Not meeting inclusion criteria (*n* = 42)

Declined to participate (*n* = 19)

Other reasons (*n* = 11)

Assessed for eligibility

(*n* = 135)

Enrolled (*n* = 63)

Started sunitinib treatment (*n* = 62)

Withdrew from sunitinib treatment before 6 mo

(*n* = 38; including 6 complete withdrawals)

Intolerance of treatment (*n* = 18)

Patient choice (*n* = 2)

Clinician choice (*n* = 11)

Disease progression (*n* = 5)

Poor performance status (*n* = 1)

Bowel obstruction (*n* = 1)

Lost to follow-up due to withdrawn consent (*n* = 6;

1 patient did not receive any trial treatment)

Assessed for primary end point (*n* = 58; 1 of the 6 patients who withdrew consent was assessable for response before withdrawal)
